# A protocol for systematic review and meta-analysis on psychosocial factors related to rehabilitation motivation of stroke patients

**DOI:** 10.1097/MD.0000000000023727

**Published:** 2020-12-24

**Authors:** Moon Joo Cheong, Byeonghyeon Jeon, Se-Eung Noh

**Affiliations:** Rare Diseases Integrative Treatment Research Institute in Wonkwang University Jangheung Integrative Medical Hospital, Jangheung-gun, Jeollanam-do, Republic of Korea.

**Keywords:** protocol, psychosocial factors, rehabilitation motivation, review, stroke

## Abstract

**Background:**

Rehabilitation motivation is more important than any other factor in terms of treatment effects among stroke patients. The goal of this study is to explore the variables related to rehabilitation motivation that affect treatment effects and analyze their effect sizes, in order to manage the psychosocial interventions required by stroke patients.

**Methods:**

Thirteen electronic databases will be searched from November to December 2020. The search terms will be composed of the disease term part (eg, “stroke”) and the intervention term part (eg, “rehabilitation motivation or rehabilitation factors related to motivation or self-efficacy or family support or rehabilitation adherence or achievement or psychosocial factors, including self-motivation, social support, psychological distress, rehabilitation adherence”). Selected studies the for systematic review and meta-analysis will include randomized, quasi-randomized, and nonrandomized controlled trials, and research programs on rehabilitation motivation; qualitative research and case studies will be excluded. The participants will be stroke patients. Two authors will independently assess each study for eligibility and risk of bias, and to extract data.

**Results:**

This study will comprehensively explore the psychosocial and physical behavioral variables related to the rehabilitation motivation of stroke patients and provide their priorities and effect sizes. In addition, we will report the magnitude of the correlation effect on the rehabilitation motivation of stroke patients according to each demographic variable.

**Conclusions:**

The conclusions of our study will provide effective evidence of psychosocial variables that influence the treatment outcomes of stroke patients.

**PROSPERO registration number:**

CRD42020207467

## 1 Introduction

“Patients can move by themselves and lead independent lives.”

The ultimate goal of all treatment is to allow stroke patients to move by themselves and achieve an independent life. To achieve this, the doctor diagnoses the patient, develops a treatment plan, and implements it.^[1]^ However, treatment effects cannot be achieved through the efforts of doctors alone. It is a sensitive work through interaction with the patient that results in the patient's will and motivation to engage in treatment.^[2]^ In particular, rehabilitation treatment is effective or discontinued not only according to the patient's rehabilitation motivation^[3]^ but also to the family's economic ability and psychological support to help the patient undergo long-term treatment.^[4,5]^ However, in the past, rehabilitation treatment from a traditional point of view focused on simply recovering the impaired function rather than prioritizing the patient's will or goal of treatment, and viewed function recovery as the main treatment effect.^[6]^ However, around the world, the concept of disability has shifted from permanent damage to the body to the possibility of activity and participation in society. Rehabilitation treatment no longer regards recovery of physical function as the goal of treatment, but has started to pay attention to the patient's return to daily routine.^[7]^ This change in perspective has made it possible to understand the cases in which patients who received rehabilitation treatments were unable to return to their daily lives even though their functional impairment had resolved.^[8]^ In addition, it was predicted that the inability to return to their daily life may occur in patients who have a low probability of cure and require long-term rehabilitation. According to Choi et al,^[9,10]^ such patients would include those with severe diseases that are caused by brain damage, such as stroke, and for which a cure is unlikely at 6 months after onset. In fact, most stroke patients have to completely or partially depend on others, and 12% to 18% of them also experience speech impairment.^[11]^

These physical impairments and perceptions of continuous rehabilitation treatment reduce the adherence of stroke patients^[12]^ and, in severe cases, lead to stopping rehabilitation.^[13]^ Stroke patients may experience anger, frustration, and depression as well as increased economic burden due to the rehabilitation treatment and family discord due to long-term treatment period.^[14,15]^ This negative emotional experience and the persistence of a disability that is difficult to resolve lowers patients’ rehabilitation motivation and may cause them to stop rehabilitation. Accordingly, among severely ill patients, especially stroke patients who need long-term rehabilitation treatment, the lower the rehabilitation motivation, the more difficult the rehabilitation treatment becomes.^[16,17]^

In addition, it is important to understand patients’ rehabilitation from a psychological point of view, and not only physical, as the patient has to leave the treatment facility and live life with a disability even when physical function is recovered. Since treatment is performed mainly focusing on functional recovery, stroke patients with a low probability of cure may face frustration in the rehabilitation process. In other words, the key to a successful rehabilitation may involve setting a realistic period of rehabilitation.^[18]^

This is not a period of rehabilitation for the recovery of function as it was before the occurrence of the disease, but a goal to return to daily life in a state of recognizing the extent of realistic recovery and accepting the persisting disability.^[19]^ However, there have been no studies investigating the factors related to the rehabilitation motivation, which is a key variable for rehabilitation outcomes and for the ultimate goal of rehabilitation, namely “return to daily life.” However, stroke studies have analyzed the relationship between self-efficacy, self-esteem, motivation-related rehabilitation,^[15]^ family support, economic status,^[20]^ and ability as an environmental factor influencing rehabilitation performance.^[21]^ However, it has become necessary to comprehensively analyze and organize these variables related to rehabilitation motivation. Therefore, this study aims to identify variables related to rehabilitation motivation in stroke patients by conducting a systematic literature review and a correlation meta-analysis, and to comprehensively organize the findings of previous studies.

For this purpose, we aim to identify factors related to “rehabilitation motivation” among stroke patients and provide important information for future rehabilitation interventions. In addition, by classifying and organizing the psychosocial variables and physical behavior factors related to the rehabilitation motivation of stroke patients, the role of psychosocial intervention methods in rehabilitation treatment in the future will be provided as basic data in the fields of medical welfare and medical humanities.

## 2 Methods and analysis

### 2.1 Study registration

The protocol for this systematic review was registered in the International Prospective Register of Systematic Reviews (PROSPERO) (registration number: CRD42020207467) on November 10, 2020. This study will involve and update a systematic review according to this protocol. This protocol will be reported in accordance with the Preferred Reporting Items for Systematic Review and Meta-Analysis Protocols 2015 statement^[22]^ and the Cochrane Handbook for Systematic Reviews of Interventions.^[23]^ If the protocol represents an amendment of a previously completed or published protocol, identify as such and list changes

### 2.2 Data sources

The following databases will be searched comprehensively from their inception to November 2020 by 2 independent researchers (MJC and BHJ): 6 English-language databases (MEDLINE via PubMed, EMBASE via Elsevier, the Cochrane Central Register of Controlled Trials, the Allied and Complementary Medicine Database via EBSCO, the Cumulative Index to Nursing and Allied Health Literature via EBSCO, and PsycARTICLES via ProQuest), 5 Korean-language databases (Oriental Medicine Advanced Searching Integrated System, Korean Studies Information Service System, Research Information Service System, Korean Medical Database, and Korea Citation Index), and 2 Chinese-language databases (China National Knowledge Infrastructure and Wanfang Data). We will also search the reference lists of the relevant articles and perform a manual search on Google Scholar to identify additional articles. We will include not only the literature published in journals but also “gray literature” such as theses and conference proceedings. There will be no language restrictions.

### 2.3 Search strategies

The search terms will be composed of the disease term part (eg, “stroke”) and the intervention term part (eg, “rehabilitation motivation or rehabilitation factors related to motivation or self-efficacy or family support or rehabilitation adherence or achievement or psychosocial factors, including self-motivation, social support, psychological distress, rehabilitation adherence”). The search strategies for the MEDLINE and EMBASE databases are shown in Table 1 and will be modified and used similarly for the other databases.

**Table 1:** Study of type according to PICO.

Criteria factor	Standard contents
Research method	RCT studies as Quantitative research method (except for the retrospective studies, retrospective study, in vivo, in vitro, case reports or studies, qualitative studies, uncontrolled trials)
Research design	RCT Studies
Purpose	It is reasonable for research purposes should be revealed.
Participants/patients	Stroke patients and there was no restriction on the sex or race, age of the participants.
Intervention/moderate variables	Factors related rehabilitation motivation
Comparison	Placebo and blank control
Outcomes	-Primary Outcomes
	The Patient Questionnaire Rehabilitation Motivation (PAREMO)
	-Secondary Outcomes
	Rehabilitation adherence Modified Barthel index
	NIHSS, SIS, SSS, SS-QOL
Data statistics	All sorts of figures, such as mean, standard deviation, *t*, *f* values, calculating effect size

### 2.4 Inclusion criteria

#### 2.4.1 Types of studies

Selected studies for systematic review and meta-analysis will include randomized controlled clinical trials, quasi-randomized controlled trials, controlled (nonrandomized) clinical trials, and research programs on rehabilitation motivation; qualitative research and case studies will be excluded.

#### 2.4.2 Types of participants

We will include studies with stroke patients. There will be no restriction on the gender, age, or race of the participants.

#### 2.4.3 Types of interventions and comparators

Studies using psychosocial variables or factors related to rehabilitation motivation will be included. We will also include studies using social behavioral variables such as socioeconomic status and family support, and individual internal and external variables related with the rehabilitation adherence or treatment. There are no comparators.

### 2.5 Types of outcome measures

#### 2.5.1 The primary outcome

The primary outcome measure is the rehabilitation motivaton, assessed with the Patient Questionnaire Rehabilitation Motivation, developed by Hafen, Jastrebow, Nubling, and Bengel (2001),^[24]^ like other rehabilitation motivation assessment tools used as a measurement and evaluation tool in each study.

#### 2.5.2 The secondary outcome

The secondary outcome measures will use tools that can be evaluated in terms of psychological factors and physical behaviors related to rehabilitation motivation.

1) Rehabilitation adherence The rehabilitation adherence assessment tool for stroke patients developed by Park (2014)^[15]^ consists of a total of 29 questions: 5 on medications, 3 on rehabilitation exercises, 3 on bedsores prevention, 2 on aspiration prevention, and 2 on health behaviors.
  Adherence measures are of 3 types^[25]^:
  (1) Patient monitoring: Patient attendance to rehabilitation sessions is monitored. For each participant, the ratio of sessions attended to scheduled sessions is calculated. Attendance has been used as an adherence measure in previous sports injury research.^[26]^
  (2) Sport injury rehabilitation adherence scale^[27]^ (Brewer, Van Raalte, Petitpas, Sklar, & Ditmar, 1995) at each physical therapy appointment, the practitioner (eg, physical therapist or athletic trainer) responsible for the rehabilitation of each participant on that day completes the sport injury rehabilitation adherence scale.
  (3) Patient self-reports of home exercise: At each rehabilitation session, patients report their degree of completion of prescribed home exercises on a scale ranging from 1 (none) to 10 (all).
2) Modified Barthel index
  The modified Barthel index developed by Austrian occupational therapists will be used to evaluate the performance of daily activities.
3) National Institutes of Health Stroke Scale, stroke impact scale, Scandinavian stroke scale, stroke specific quality of life scale

### 2.6 Study selection

**Figure 1:**
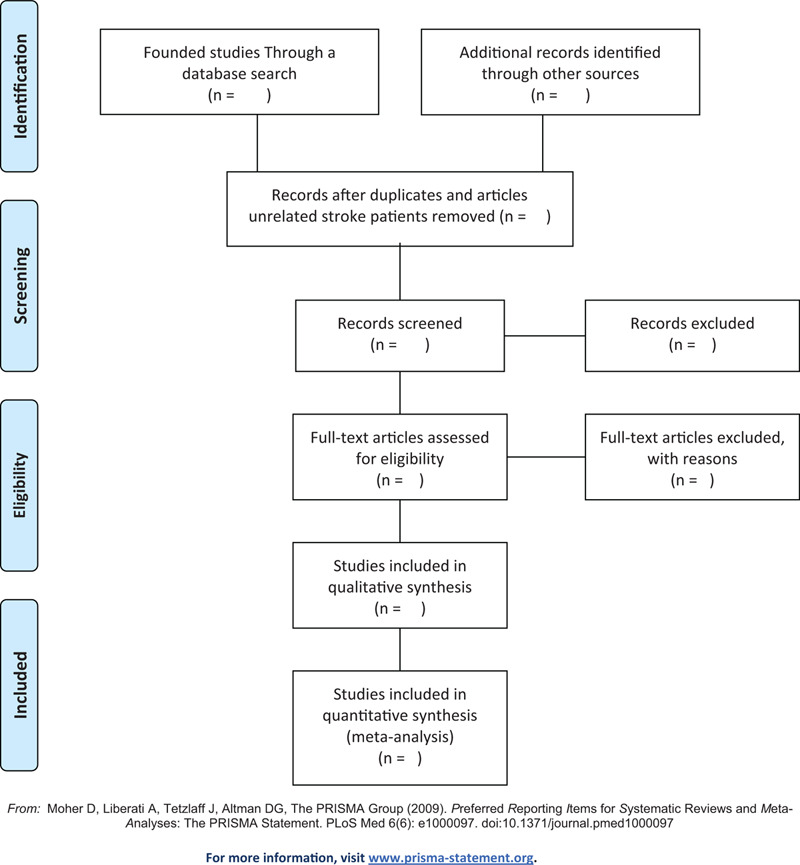
PRISMA flow diagram. PRISMA = preferred reporting items for systematic review and meta-analysis.

The study selection will be conducted by 2 independent researchers, MJC and BHJ, according to the above selection criteria (Table 1). After removing duplicates, we will select and review the titles and abstracts of the searched studies for relevance, and will then evaluate the full texts of the selected studies for eligibility. Any disagreement on study selection will be resolved through discussion with other researchers. The literature selection process will be reported in accordance with the preferred reporting items for systematic review and meta-analysis guidelines^[28]^ (Fig. 1).

### 2.7 Data extraction

The extracted studies will include the first author's name, year of publication, country, paper title, sample size and number of dropouts, age, and gender of participants, details of intervention and comparison, research design, measurement tools, independent, dependent, mediated, and control variables, and sub-factors related to rehabilitation motivation. For example, a psychosocial variable is extracted as an intervention variable related to the rehabilitation motivation, which is an outcome variable, and then classified as a psychological or social variable, and the sub-variables include factors that reduce and improve rehabilitation motivation. Subsequently, the variables related to rehabilitation motivation in each study will be classified and structured as factors (eg, depression as a psychological risk factor, resilience as a protective factor, economic burden as a risk factor, and family support as a protective factor). The extracted data will be recorded using Excel 2016 (Microsoft, Redmond, WA) and will be shared among researchers using Dropbox (Dropbox, Inc., CA) folders. We will contact the corresponding authors of the included studies via email to request additional information if the data are insufficient or ambiguous.

### 2.8 Quality assessment

Two independent researchers, MJC and BHJ, will assess the methodological quality of the included studies and the quality of the evidence for each main finding. Discrepancies will be resolved through discussion with other researchers. The methodological quality of the included studies will be assessed using the Cochrane Collaboration risk-of-bias tool.^[29]^ We will assess random sequence generation, allocation concealment, blinding of participants and personnel, blinding of outcome assessments, incomplete outcome data, selective reporting, and other biases for each included study. Each domain will be categorized into 1 of 3 groups: “low risk,” “unclear,” or “high risk.” Each evaluation will be recorded in an Excel 2016 spreadsheet and will be shared among researchers using Dropbox (Dropbox, Inc.) folders. The evaluated results will be presented in a full review using Review Manager version 5.3 (Cochrane, London, UK). The results of the quality of evidence will be presented through a summary-of-findings table. The evaluation process will be shared and discussed by researchers.

### 2.9 Data synthesis and analysis

Data synthesis and analysis will be performed using Review Manager Version 5.3 (Cochrane) and Excel 2016, and files will be shared among researchers using Dropbox (Dropbox, Inc.) folders. Descriptive analyses of the details of participants, interventions, and outcomes will be conducted for all included studies. A quantitative synthesis will be performed if there are studies using the same types of intervention, comparison, and outcome measures. The collected data will be analyzed in 2 stages by first synthesizing and analyzing the data according to the systematic review process, and then classifying the studies with figures that can be meta-analyzed. In the first stage, a systematic review aims to comprehensively organize and analyze psychosocial variables related to the rehabilitation motivation of stroke patients. A Study on the effect of psychological intervention in the recovery of stroke patients and exploring individual psychological and environmental variables, such as support for rehabilitation motivation of stroke patients. Therefore, this study will be classified and coded to “author (year of publication),” “subjects (patients),” psychosocial factors and sub-factors that affect the rehabilitation motivation of stroke patients, measurement tools of rehabilitation motivation and research methods, research procedures, and research results. We will synthesize and analyze each paper in this way. In the second step, the psychosocial factors related to the rehabilitation motivation of stroke patients used in the meta-analysis will be systematized through discussions and reviews among researchers.

The framework of the analysis category will be nominated and coded based on the following items in order to calculate the size of the correlation for each study. The data coding for the meta-analysis will be as follows. First, the psychosocial variables related to the rehibiliatation of stroke patients will be classified as psychological or social variables. Second, psychological and social variables will be divided into risk factors having a negative correlation and protective factors having a positive correlation with rehabilitation motivation. Third, the sub-variables of risk factors and protection factors will be synthesized by identifying the correlation code in studies on psychosocial variables in stroke patients, reviewing the theoretical background, and classifying each variable into an easy frame for analysis. After that, we will analyze the overall publishing bias, homogeneity verification, overall correlation effect size analysis, and correlation effect size between all factors related rehabilitation motivation. The correlation effect size will be analyzed using Fisher *z*^[30]^ (.1 for small effect size, .3 for medium effect size, and .5 for large effect size) by checking the correlation coefficient in the 95% confidence interval.

Heterogeneity between the studies in terms of effect measures will be assessed using both the chi-squared test and the *I*-squared statistic. We will consider *I*-squared values greater than 50% and 75% indicative of substantial and high heterogeneity, respectively. In the meta-analyses, a random effects model will be used when the heterogeneity is significant (*I*-squared value > 75%), while a fixed effects model will be used when the heterogeneity is non-significant. A fixed effects model will be also used when the number of studies included in the meta-analysis is very small, where inter-study variance estimates have poor accuracy.^[31]^ When it is considered that the heterogeneity is too high for the results to be synthesized (*I*-squared value > 75%), a subgroup analysis will be conducted as follows to determine the cause of heterogeneity.

### 2.10 Assessing the quality of the body evidence

The quality of the evidence was assessed using the Grading of Recommendations, Assessment, Development, and Evaluation,^[32]^ which was rated according to the following 5 categories: risk of bias, imprecision, inconsistency, indirectness, and other factors such as publication bias.^[33]^

### 2.11 Subgroup analysis

If heterogeneity is evaluated as significant (*I*-squared value > 75%) and the necessary data are available, we will conduct a subgroup analysis to account for the heterogeneity. A subgroup analysis will be conducted according to the following criteria:

(1) the stroke rehabilitation period,
(2) the hospital stay period,
(3) demographic variables, and
(4) socioeconomic status.

### 2.12 Sensitivity analysis

To identify the robustness of the meta-analysis result, we will perform sensitivity analyses by determining the effects of excluding

(1) studies with high risks of bias,
(2) studies with missing data, and
(3) outliers.

### 2.13 Assessment of reporting bias

If there are more than 10 trials included in the analysis, reporting biases such as publication bias will be assessed using funnel plots. When reporting bias is implied by funnel plot asymmetry, we will attempt to explain possible reasons.

## 3 Ethics and dissemination

Ethical approval will not be needed because the data used in this systematic review will not include individual patient data and there will be no concerns regarding privacy. The results will be disseminated by the publication of a manuscript in a peer-reviewed journal and/or presentation at a relevant conference.

## 4 Discussion

Rehabilitation treatment for stroke patients is not performed over a short period of time, and the concept of cure does not apply; thus, it is considered that patients are in rehabilitation for a lifetime (Kwon et al, 2003).^[34]^ As such, rehabilitation treatment requires a long period of time, and it is difficult to expect a satisfactory rehabilitation effect without the patient's active participation, a clear goal setting for the rehabilitation period, and the economic support of the family. Even if being an economically rich or with competent therapist, rehabilitation is likely to be stopped if the patient has no or low rehabilitation motivation. In particular, stroke patients show decreased willingness to rehabilitate as well as feelings of frustration and anger when they are not in the shape or situation they expect at a particular stage of recovery through rehabilitation. Therefore, it will be useful to design effective therapeutic interventions to identifying the variables that affect the rehabilitation motivation of stroke patients. However, until now, systematic searches for such variables have not been conducted. Therefore, in this study, we aim to explore the variables related to the rehabilitation motivation, one of the major factors in the treatment of stroke patients. We believe the results of this systematic review will help clinicians optimize treatment protocols for stroke patients. It is also expected that social welfare and health policy makers will be able to identify areas in the public health setting that require intervention to improve treatment for stroke patients.

## Author contributions

**Conceptualization:** Moon Joo Cheong, Byeonghyeon Jeon.

**Data curation:** Moon Joo Cheong.

**Formal analysis:** Moon Joo Cheong.

**Funding acquisition:** Se-Eung Noh.

**Investigation:** Moon Joo Cheong.

**Methodology:** Moon Joo Cheong.

**Project administration:** Moon Joo Cheong.

**Resources:** Se-Eung Noh.

**Supervision:** Moon Joo Cheong, Se-Eung Noh.

**Writing – original draft:** Moon Joo Cheong.

**Writing – review & editing:** Moon Joo Cheong, Byeonghyeon Jeon.

## References

[1] Maclean N Pound P Wolfe C Qualitative analysis of stroke patients” motivation for rehabilitation. BMJ 2000;321:1051–4. 11053175 10.1136/bmj.321.7268.1051 PMC27512

[2] Kaplan SH Greenfield S Ware JE Assessing the effects of physician-patient interactions on the outcomes of chronic disease. Med Care 1989;27:S110–27. 2646486 10.1097/00005650-198903001-00010

[3] Duncan PW Zorowitz R Bates B Management of adult stroke rehabilitation care: a clinical practice guideline. Stroke 2005;36:e100–43. 16120836 10.1161/01.STR.0000180861.54180.FF

[4] Kim Y Given BA Quality of life of family caregivers of cancer survivors: across the trajectory of the illness. Cancer 2008;112 (S11):2556–68. 18428199 10.1002/cncr.23449

[5] Visser-Meily A Post M Gorter JW Rehabilitation of stroke patients needs a family-centred approach. Disabil Rehabil 2006;28:1557–61. 17178619 10.1080/09638280600648215

[6] Palmer S Glass TA Family function and stroke recovery: a review. Rehabil Psychol 2003;48:255–65.

[7] Szilvia Geyh S Cieza A Schouten J ICF core sets for stroke. J Rehabil Med 2004;36:135–41. 10.1080/16501960410016776 15370761

[8] Indredavik B Bakke F Slørdahl SA Stroke unit treatment improves long-term quality of life: a randomized controlled trial. Stroke 1998;29:895–9. 9596231 10.1161/01.str.29.5.895

[9] Choi ES Lee EN Cho JL The mediating effect of resilience on depression and rehabilitation motivation in stroke patients. J Muscle Joint Health 2016;23:19–27.

[10] Balaam M Egglestone SR Fitzpatrick G Motivating mobility: designing for lived motivation in stroke rehabilitation. In: Proceedings of the SIGCHI Conference on Human Factors in Computing Systems 2011 3073–82.

[11] Leys D Atherothrombosis: a major health burden. Cerebrovasc Dis 2001;11: Suppl 2: 1–4. 10.1159/000049137 11316915

[12] Mosca L Effectiveness-based guidelines for the prevention of cardiovascular disease in women—2011 update: a guideline from the American Heart Association. J Am Coll Cardiol 2011;57:1404–23. 21388771 10.1016/j.jacc.2011.02.005 PMC3124072

[13] Thomson K Commercial gaming devices for stroke upper limb rehabilitation: the stroke survivor experience. J Rehabil Assist Technol Eng 2020;7:2055668320915381. 10.1177/2055668320915381 PMC8855408 35186319

[14] Chang FH Lin YN Liou TH XXXX. Disabil Rehabil 2020 1–8.

[15] Park AS Ko E Influences of rehabilitation motivation, self-efficacy and family support on rehabilitation adherence in stroke patients. J Korean Biol Nurs Sci 2017;19:113–22.

[16] Park YS Kweon SS Factors affecting stroke patients” rehablilitation motivation. Korean Public Health Research 2002;28:21–30.

[17] Korean Academy of Nursing Science Conference, Lee H-J Lee M-S Factors Affecting the Reactivation Period of Stroke Patients. 2004;125–1125.

[18] Kong H-K Lee H-J Effect of empowerment program on rehabilitation motivation, depression, activities of daily living among the patients with stroke. Korean J Adult Nurs 2008;20:406–17.

[19] Moon J-Y Cho B-H Relationships among rehabilitation motivation, perceived stress and social support in stroke survivors. Korean J Rehabil Nurs 2011;14:24–31.

[20] Jun H-J Kim K-J Chun I-A The relationship between stroke patients” socio-economic conditions and their quality of life: the 2010 Korean community health survey. J Phys Therapy Sci 2015;27:781–4. 10.1589/jpts.27.781 PMC4395714 25931730

[21] Medin J Barajas J Kerstin Ekberg K Stroke patients” experiences of return to work. Disabil Rehabil 2006;28:1051–60. 16950735 10.1080/09638280500494819

[22] Shamseer L Moher D Clarke M Preferred reporting items for systematic review and meta-analysis protocols (PRISMA-P) 2015 elaboration and explanation. BMJ 2015;350:g7647. 25555855 10.1136/bmj.g7647

[23] The Cochrane Collaboration. In: Higgins JPT, Green S, eds. Cochrane Handbook for Systematic Reviews of Interventions Version 5.1.0, 2011. Available at: http://handbook.cochrane.org/ [access date July 2018).

[24] Hafen KJRJ Entwicklung eines Patientenfragebogens zur Erfassung der Reha-Motivation (PAREMO). Die Rehabil 2001;40:3–11. 10.1055/s-2001-12136 11253752

[25] Brewer B Vanraalte JL Cornelius AE Psychological factors, rehabilitation adherence, and rehabilitation outcome after anterior cruciate ligament reconstruction. Rehabil Psychol 2000;45:20–37.

[26] Byerly PN Worrell T Gahimer J Rehabilitation compliance in an athletic training environment. J Athletic Training 1994;29:352. PMC1317812 16558300

[27] Joanne MD Cognitive appraisal, emotional adjustment, and adherence to rehabilitation following knee surgery. J Sport Rehabil 1995;41:23–30.

[28] Liberati A Altman DG Tetzlaff J The PRISMA statement for reporting systematic reviews and meta-analyses of studies that evaluate health care interventions: explanation and elaboration. Ann Intern Med 2009;151:W65–94. 19622512 10.7326/0003-4819-151-4-200908180-00136

[29] Higgins JPT Altman DG The Cochrane Collaboration. Chapter 8: assessing risk of bias in included studies. In: 398 Higgins J, Green S, Eds, Cochrane Handbook for Systematic Reviews of Interventions Version 5.1.0, 2011. 399. Available at: http://www.cochrane-handbook.org.

[30] BORENSTEIN, Michael, et al. Effect Sizes for Continuous Data. The Handbook of Research Synthesis and Meta-analysis, 2009, 2: 221-235.

[31] Borenstein M Hedges LV Higgins JPT A basic introduction to fixed- effect and random-effects models for meta-analysis. Res Synth Methods 2011;1:97–111. 10.1002/jrsm.12 26061376

[32] Comondore VR Devereaux PJ Zhou Q Quality of care in for-profit and not-for-profit nursing homes: systematic review and meta-analysis. BMJ 2009;339:b2732. 19654184 10.1136/bmj.b2732 PMC2721035

[33] Howard B Helfand M Schünemann HJ GRADE guidelines: 3. Rating the quality of evidence. J Clin Epidemiol 2011;64:401–6. 21208779 10.1016/j.jclinepi.2010.07.015

[34] Kwon EH A Study on the Rehabilitation Motive of Stroke Patients: Focused on Individual and Family Support Factors (Master's Thesis). Seoul: University of Ewha Womans; 2003.

